# Are Asymmetry-Focused Training Interventions More Effective than General Programs in Reducing Interlimb Asymmetries and Improving Athletic Performance? A Systematic Review

**DOI:** 10.1186/s40798-026-01119-5

**Published:** 2026-10-13

**Authors:** Antonio Martos-Arregui, Iago Rojas-Cepero, Xing Zhang, Amador García-Ramos, Francisco Javier Rojas-Ruiz, Mar Cepero-González

**Affiliations:** 1 Department of Physical Education and Sport, Faculty of Sport Sciences, University of Granada, Camino de Alfacar, 21, 18071 Granada, Spain; 2 Department of Sports Sciences and Physical Conditioning, Faculty of Education, Universidad Católica de La Santísima Concepción, Concepción, Chile; 3 Department of Didactics of Musical, Plastic and Corporal Expression, Faculty of Educational Sciences, University of Granada, Granada, Spain

**Keywords:** Interlimb asymmetries, Physical performance, Training intervention, Athletic population

## Abstract

**Background:**

Interlimb asymmetries (ILA) have been associated with an increased risk of injury and decreased athletic performance. However, despite the growing number of training interventions targeting ILA, their comparative effectiveness relative to general strength and conditioning approaches has not yet been systematically reviewed.

**Objective:**

This systematic review aimed to compare the effectiveness of asymmetry-focused training interventions versus general training programs in reducing ILA and improving physical performance in athletic populations. Specifically, it sought to compare individualized training programs that first identify the weaker limb during pre-testing and subsequently prescribe a greater training stimulus to that limb with general training protocols that apply the same training stimulus to both limbs, as well as unilateral approaches with bilateral training strategies.

**Methods:**

Following PRISMA 2020 guidelines, a systematic search was conducted in Web of Science, PubMed, and SPORTDiscus up to April 2025. Eligible studies involved healthy athletes (aged 12–30 years) who completed ≥4-week lower-limb training interventions that either applied a greater stimulus to the weaker limb (e.g., starting sets with the weaker side or adding volume) or used unilateral training approaches that applied the same training stimulus to both limbs regardless of baseline ILA values. To be included, studies were required to assess both pre-to-post changes in ILA and at least one physical performance outcome. The risk of bias was assessed using a modified Cochrane tool.

**Results:**

Eleven studies comprising 297 athletes met the inclusion criteria. Reductions in ILA were more consistent in asymmetry-focused and unilateral training groups compared to general and bilateral training groups, respectively. Most interventions also led to improvements in physical performance, with asymmetry-focused and unilateral protocols typically producing greater gains in single-limb tasks, although bilateral training showed more favourable effects in bilateral tasks. However, variability in training protocols and outcome measures across studies limits the generalizability of these findings.

**Conclusion:**

Asymmetry-focused strategies, such as starting unilateral exercises with the weaker limb or increasing its training volume, appear to be more effective for reducing ILA than non-individualized unilateral training. These approaches are practical, safe, and potentially beneficial in athletic settings. However, further high-quality research is needed to confirm their superiority over non asymmetry-focused unilateral or bilateral training and to establish clear guidelines for their implementation.

*Registration *PROSPERO-CRD420251023232.

**Supplementary Information:**

The online version contains supplementary material available at https://doi.org/10.1186/s40798-026-01119-5.

## Key Points

1. Asymmetry-focused training strategies, such as prioritizing the weaker limb, may reduce interlimb asymmetries and enhance performance more effectively than general programs.
2. Asymmetry-focused training strategies are practical, low-risk, and easy to apply in athletic settings.
3. The effectiveness of asymmetry-focused training depends on accurately determining the stronger and weaker limb and using performance-based assessments aligned with the task being trained or assessed.

**Supplementary Information:**

The online version contains supplementary material available at https://doi.org/10.1186/s40798-026-01119-5.

## Introduction

Interlimb asymmetries (ILA) in the lower limbs refer to measurable performance differences or imbalances between the left and right legs. These asymmetries are typically identified using a variety of physical tests, such as change of direction (COD) tasks, resistance training exercises, and jump-based assessments [1, 2]. Importantly, both the direction and magnitude of ILA are specific to the task and metric used [3, 4], and they may fluctuate over time [5]. Although thresholds of 10–15% are commonly used to identify meaningful ILA, establishing a universal cut-off remains challenging due to variability across tasks, metrics, and populations [1, 4]. Moreover, asymmetries should exceed the coefficient of variation (CV) of the test to be considered real and not simply attributable to measurement error [6, 7]. Nevertheless, the importance of addressing ILA through targeted training interventions is widely recognized [8, 9]. In recent years, there has been growing interest in developing strategies to reduce ILA, aiming to enhance athletic performance and potentially mitigate injury risk [10–14]. Despite this increased attention, the literature remains fragmented regarding the characteristics and effectiveness of these interventions, highlighting the need for a comprehensive synthesis of current evidence.

To date, only two systematic reviews have specifically examined the effects of physical training on ILA [8, 9]. Bettariga et al. [9] reported small to moderate effects of both unilateral and bilateral strength training on reducing ILA in different tests such as the single-leg countermovement jump (SL-CMJ) and single-leg horizontal jump (SL-HJ). However, their meta-analysis did not explicitly differentiate between interventions specifically designed to reduce ILA and those with a more general training focus. More recently, Sun et al. [8] found that unilateral plyometric training reduced ILA in jump-based assessments compared to bilateral plyometric or complex training interventions. Nonetheless, the review focused exclusively on plyometric training, thereby excluding other commonly used training interventions such as eccentric overload training [14], strength training [15], or the combination of strength and plyometric training [16]. Furthermore, the authors did not evaluate whether the training programs were intentionally designed to reduce ILA, and they overlooked the potential impact of applying different training stimuli to each limb on training outcomes.

There is growing research interest in identifying targeted strategies that may be more effective in reducing ILA. One promising approach involves applying a greater training stimulus to the weaker limb. This strategy may lead to greater improvements compared to bilateral training alone [12, 13, 17], or to unilateral training that does not specifically focus on the weaker side [10, 14, 18]. As highlighted by Maloney [19], the weaker limb represents a “window for development,” and enhancing its capacity may contribute not only to ILA reduction but also to overall performance improvement. However, some findings indicate that emphasizing the weaker limb may also lead to performance gains [18]. Furthermore, unilateral training has gained increasing attention as a strategy to address ILA [8]. Because unilateral training requires each limb to generate force independently, it minimizes compensation by the stronger leg and may promote more balanced adaptations [13]. In fact, some evidence suggests that unilateral protocols may be more effective than bilateral training alone in addressing ILA [11, 20]. Nonetheless, there is also evidence showing that both unilateral and bilateral training can produce comparable improvements in performance outcomes [16].

Despite the range of interventions investigated to date, it remains unclear which types of training are most efficient for reducing ILA in athletic populations. Clarifying this could provide valuable practical guidance for coaches and practitioners seeking to implement more targeted and individualized training strategies. Moreover, understanding which approaches yield the most favorable outcomes may encourage future research focused on the development of specific asymmetry-reduction protocols and their influence on athletic performance. Therefore, this systematic review aims to evaluate the effectiveness of different training approaches in reducing ILA and enhancing physical performance in athletic populations. Specifically, it first compares programs explicitly designed based on baseline asymmetry values to general training interventions that do not consider individual ILA in their prescription. Second, it examines whether unilateral training programs, despite not being tailored to initial ILA levels, are more effective than bilateral approaches in decreasing asymmetries.

## Methods

### Protocol and Registration

This systematic review was conducted in accordance with the 2020 Preferred Reporting Items for Systematic Reviews and Meta-Analyses (PRISMA) guidelines [21]. The review protocol was prospectively registered in the International Prospective Register of Systematic Reviews (PROSPERO; registration number: CRD420251023232).

### Search Strategy

A comprehensive search strategy was developed using combinations of terms related to training interventions, physical performance outcomes, and ILA, which were specifically combined using the Boolean operator AND. Additional search refinements were made using the operator OR to expand the scope within each category. The search was conducted in Web of Science, PubMed, and SPORTDiscus, covering studies from the inception of each database until April 14, 2025. The syntax was adapted for each database and applied to title, abstract, and keyword search fields. The full search strategy used in each database is provided in Supplementary Material 1. A second search was conducted by reviewing the reference lists of the studies that were included after full-text screening. Furthermore, a third search was performed using the Related articles feature in Google Scholar for each of the studies selected for the systematic review. Abstracts, editorials, opinion pieces, conference proceedings, and academic theses were not considered for inclusion.

### Eligibility Criteria

A PICO framework was applied to define the inclusion and exclusion criteria as follows:

- P (Population): Healthy youth and adult athletes of both sexes, aged between 12 and 30 years, with no injuries or diagnosed medical conditions.
- I (Intervention): Training interventions targeting the lower limbs and lasting at least four weeks that either applied a greater training stimulus to the limb exhibiting baseline asymmetries, or employed unilateral training approaches.
- C (Comparison): Bilateral training programs that did not account for ILA in exercise prescription, unilateral training protocols using different approaches and control groups.
- O (Outcomes): ILA during performance-based tests and athletic performance variables assessed before and after the intervention.

Only peer-reviewed studies published in English or Spanish were eligible for inclusion. Interventions that applied a greater training stimulus to the limb exhibiting baseline asymmetries were defined as those studies in which ILA were assessed prior to the intervention to identify the stronger and weaker limb. In this review, the stronger limb refers to the limb demonstrating superior performance in the assessed task, whereas the weaker limb refers to the contralateral limb, regardless of self-reported limb preference. A greater training stimulus was intentionally directed to one of the limbs during unilateral exercises (e.g., by starting sets with one of the limbs or increasing its training volume). Studies assessing ILA exclusively through isokinetic dynamometry were excluded, as this type of assessment requires specialized laboratory equipment that is not commonly available in most sports clubs or training facilities, limiting its ecological validity and practical applicability [22, 23].

### Study Selection

The identification and screening processes were conducted by the lead author (AMA) in coordination with the co-authors AGR and XZ. All records identified through the search strategy were uploaded to Rayyan (Rayyan Systems Inc., Cambridge, MA, USA) for screening management. Duplicate entries were removed by the lead author (AMA) prior to the screening process. Subsequently, titles and abstracts were reviewed to assess potential eligibility. Finally, full-text articles were retrieved for those studies deemed relevant. Any discrepancies arising during the screening process were discussed between the two reviewers and, when necessary, resolved by a third reviewer (AGR).

### Data Extraction

One reviewer (AMA) carried out the data extraction, which was subsequently verified by another reviewer (AGR). Any discrepancies were discussed and resolved through consensus among all authors. For all studies that met the inclusion criteria, relevant data were systematically extracted into an Excel spreadsheet. The extracted variables included: study characteristics (study identification and participants); assessment of ILA (metrics and limb dominance); description of training interventions; and changes in ILA and performance variables.

### Risk of Bias Assessment

Risk of bias was assessed using a modified version of the Cochrane Collaboration's tool for evaluating bias in randomized studies [24]. Given that blinding of participants and personnel is not feasible in supervised exercise interventions, the performance bias (blinding of participants and personnel) domain was excluded. An additional domain other bias was included to evaluate relevant factors within the context of sports science, specifically whether participants underwent familiarization with the testing and training protocols, and whether validated measurement instruments were employed. The final domains evaluated were: random sequence generation, allocation concealment, blinding of outcome assessment, incomplete outcome data, selective reporting, and other sources of bias. The assessment was conducted using RevMan Web [25], the online version of the Review Manager software. Each study was categorized according to its overall risk of bias as follows: low risk, moderate risk, or high risk.

## Results

### Study Selection

**Fig. 1:**
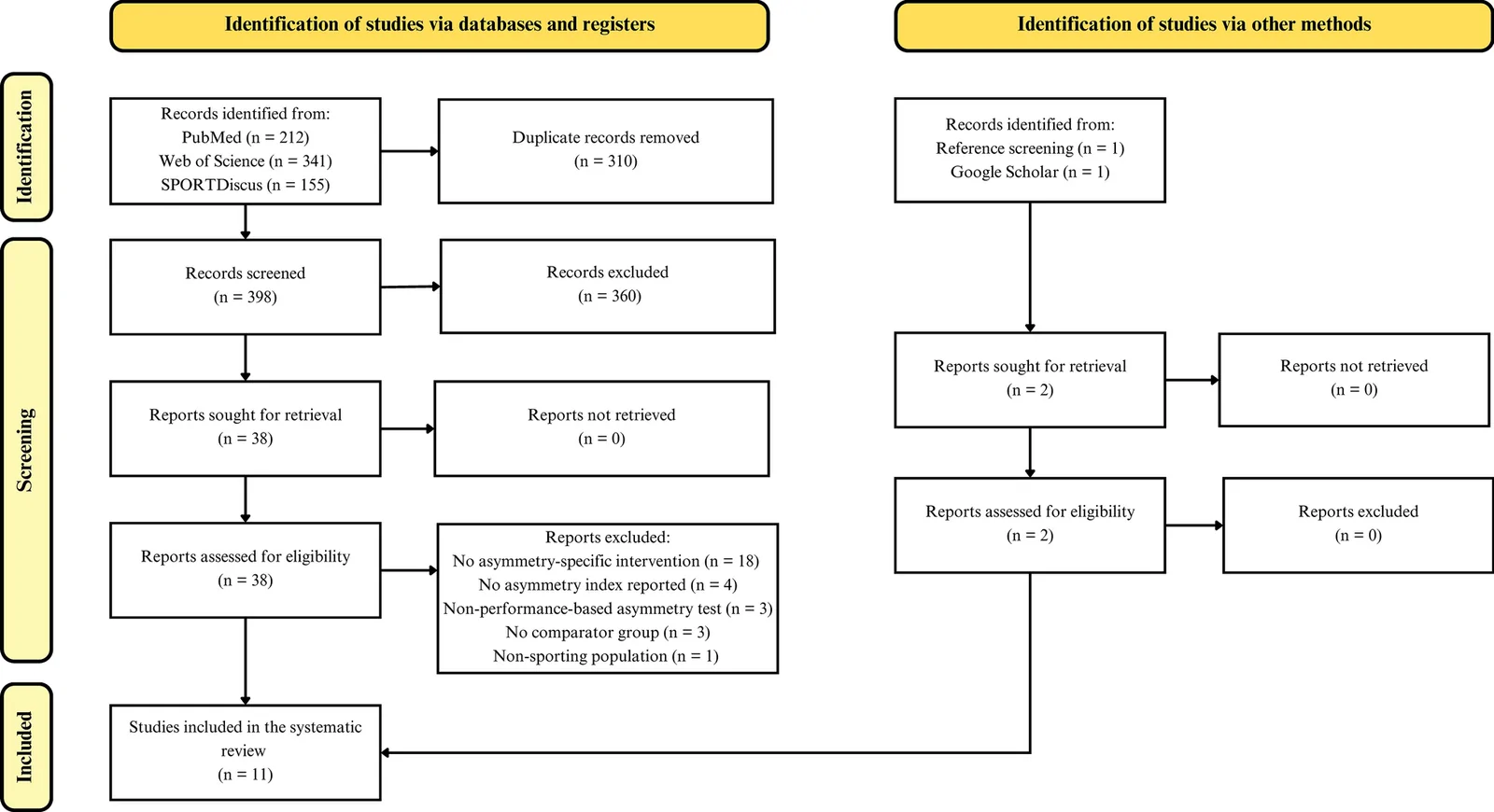
Study selection flow diagram

The flowchart outlining the study selection process is shown in Fig. 1. The initial database search yielded a total of 708 records. After removing duplicates, 398 studies remained for title and abstract screening, which led to the exclusion of 360 articles that did not meet the inclusion criteria. Subsequently, 38 full-text articles were assessed for eligibility. Of these, 29 studies were excluded for reasons such as no asymmetry-specific intervention, no asymmetry index reported, non-performance-based asymmetry test, no comparator group or non-sporting population. Two additional studies were retrieved through reference screening and related articles in Google Scholar. Finally, 11 studies met all inclusion criteria and were included in the present systematic review.

### Study Characteristics

The studies analyzed in this review were conducted between 2017 and 2025 and focused on athletic populations, with participants ranging from youth to adult athletes. Most studies involved male athletes, although one study did not report the sex of the participants [26]. The level of participants varied across studies and included elite, highly trained, university-level, and amateur athletes. A variety of sports were represented, including soccer, basketball, karate, and handball. Sample sizes varied across studies (from 18 to 66 participants), with a total of 297 participants. Two studies [10, 14] conducted by the same research group appeared to use the same sample; therefore, these participants were only counted once in the total sample size.

### Description of the Included Studies

The included studies are summarized in Table 1 and grouped into two main categories based on the type of intervention applied: interventions that compare different training stimuli between limbs, such as adjusting volume or prioritization for one leg over the other, and interventions that compare unilateral versus bilateral training approaches. Within each category, studies are presented in chronological order according to their year of publication.

**Table 1:** Summary of the study characteristics, assessment of interlimb asymmetries (ILA), training intervention and changes in ILA and performance variables reported by the studies included in this review

Study	Study characteristics (participants and group allocation)	Assessment of ILA (metrics, limb dominance assessment and equation)	Training intervention	Changes in ILA and performance variables
**Interventions Applying Different Training Stimuli Between Limbs**				
Gonzalo-Skok et al. [17]	*n* = 18 (M) 16.8 ± 1.9 years Elite basketball players Uni G (starting leg = weak leg): *n* = 9 Bil G: n = 9	**Test and metrics**: Incremental Uni SQ test: ConPeak (W) **Dominance**: Strong and weak legs were assessed at the unilateral SQ pre-test **Equation**: Limb Symmetry Index 1 = (ConPeak worse leg / ConPeak better leg) × 100	6 weeks (2 sessions per week) **Uni G:** Uni 90º SQ (3 × until ConPeak < 10%) Uni DJ at 0.25 m (2 × 5) Uni CMJ (2 × 5) **Bil G:** Bil 90º SQ (3 × ConPeak<10%) Bil DJ at 0.5 m (2 × 5) Bil CMJ (2 × 5) *Between-set rest:* 3 min	**Uni G:** ↑ Uni SQ ILA; CMJ; 180º COD (left and right leg); 5-m, 15-m and 25-m sprint; Uni SQ (right and left leg); Bil SQ; SQ bilateral deficit = V-cut COD
				**Bil G:** ↑ CMJ; 180º COD (right leg); 5-m, 15-m and 25-m Sprint; Uni SQ (right and left leg); Bil SQ = Uni SQ ILA; V-cut COD; 180º COD (left leg); SQ bilateral deficit
				**Between-group comparisons:** Uni G > Bil G: Uni SQ ILA; CMJ; 180º COD (left leg); Uni SQ (right and left leg); SQ bilateral deficit
Gonzalo-Skok et al. [10]	*n* = 35 (M) 15.4 ± 0.7 years U-17 soccer players SVW: *n* = 10 DVW: *n* = 11 SVS: *n* = 14	**Test and metrics**: SL-HJ: Jump distance (cm) SL-THJ: Jump distance (cm) SL-CMJ: Jump height (cm) **Dominance**: Weaker leg = leg with worse results in the majority of 3 unilateral tests **Equation**: Percentage difference = (100 / Maximum × Minimum) × − 1 + 100	10 weeks (1 session per week) Lateral squat using conical pulley (speed:force ratio 1–3 out of 4; 2 × 6–10 reps) *Between-legs rest:* 30 s *Between-set rest:* 3 min	**SVW:** ↑ SL-THJ (strong, weak, right, and left leg); SL-CMJ (weak and right leg); CMJ = SL-HJ ILA; SL-THJ ILA; SL-CMJ ILA; SL-HJ (strong, weak, right, and left leg); SL-CMJ (strong and left leg)
				**DVW:** ↑ SL-THJ ILA; SL-THJ (strong, weak, right, and left leg); SL-CMJ (weak leg) = SL-CMJ ILA; SL-HJ (strong, weak, right, and left leg); SL-CMJ (strong, right, and left leg); CMJ ↓ SL-HJ ILA
				**SVS:** ↑ CMJ = SL-THJ ILA; SL-CMJ ILA; SL-HJ (strong, weak, right, and left leg); SL-THJ (weak, right, and left leg); SL-CMJ (strong, weak, right, and left leg) ↓ SL-HJ ILA; SL-THJ (strong leg)
				**Between-group comparisons:** SVW and DVW > SVS: SL-HJ (right leg); SL-THJ (strong, weak, and right leg) SVW > SVS: SL-HJ ILA; SL-CMJ (right leg) DVW > SVW and SVS: SL-THJ ILA DVW > SVS: SL-THJ (left leg)
Moreno-Azze et al. [14]	*n* = 35 (M) 15.4 ± 0.7 years U-17 soccer players SVW: *n* = 10 DVW: *n* = 11 SVS: *n* = 14	**Test and metrics**: Lateral SQ (1 × 10): ConMean (W), EccMean (W), ConPeak (W), EccPeak (W) **Dominance**: Weaker leg = leg with worse results in the majority of 3 unilateral tests **Equation**: Percentage difference = (100 / Maximum × Minimum) × − 1 + 100	10 weeks (1 session per week) Lateral squat using conical pulley (speed:force ratio 1–3 out of 4; 2 × 6–10 reps) *Between-legs rest:* 30 s *Between-set rest:* 3 min	**SVW**: ↑ ConMean ILA; EccMean ILA; ConPeak ILA; ConMean (strong and weak leg); EccMean (strong and weak leg); ConPeak (strong and weak leg); EccPeak (strong and weak leg) = EccPeak ILA
				**DVW**: ↑ ConMean (strong and weak leg); EccMean (strong and weak leg); ConPeak (strong and weak leg); EccPeak (strong and weak leg) = ConMean ILA; EccMean ILA; ConPeak ILA; EccPeak ILA
				**SVS**: ↑ ConMean (strong and weak leg); EccMean (strong and weak leg); ConPeak (strong and weak leg); EccPeak (strong and weak leg) = ConMean ILA; EccMean ILA; ConPeak ILA; EccPeak ILA
				**Between-group comparisons:** DVW > SVW: EccPeak ILA; ConMean (strong and weak leg); ConPeak (strong leg) DVW > SVS: EccPeak ILA; ConMean (strong and weak leg); EccMean (strong leg); EccPeak (strong and weak leg) SVW > SVS: ConPeak ILA; ConMean (strong and weak leg); EccMean (strong and weak leg); ConPeak (weak leg); EccPeak (strong and weak leg)
Moreno-Azze et al. [18]	*n* = 45 (M) 15.5 ± 0.9 years U-17 soccer players SVW: *n* = 14 DVW: *n* = 15 SVS: *n* = 16	**Test and metrics**: SL-HJ: Jump distance (cm) SL-THJ: Jump distance (cm) SL-CJM: Jump height (cm) SL-LJ: Jump distance (cm) 180º COD: time (s) Lateral SQ (2 × 10): ConMean (W), EccMean (W) **Dominance**: Stronger leg = leg with better results in most jump tests **Equation**: Percentage difference = (100 / Maximum × Minimum) × − 1 + 100	10 weeks (1 session per week) Lateral SQ on vibration platform (30HZ; 1 × 6–10 reps) Lateral SQ using conical pulley (speed:force ratio 1–3 out of 4; 2 × 6–10 reps) Single-leg side horizontal jump (2 × 4–6 reps)	**SVW:** ↑ SL-HJ (strong and weak leg); SL-THJ (strong and weak leg); SL-LJ (strong and weak leg); CMJ; SL-CMJ (strong leg); COD (weak leg); ConMean (strong and weak leg); EccMean (strong and weak leg) = SL-HJ ILA; SL-LJ ILA; COD ILA; ConMean ILA; EccMean ILA; SL-CMJ (weak leg); COD (strong leg) **↓** SL-THJ ILA; SL-CMJ ILA
				**DVW:** ↑ EccMean ILA; SL-HJ (weak leg); SL-THJ (strong and weak leg); SL-LJ (weak leg); CMJ; SL-CMJ (strong and weak leg); COD (weak leg); ConMean (strong leg); EccMean (strong leg) = SL-HJ ILA; SL-LJ ILA; COD ILA; ConMean ILA; SL-HJ (strong leg); SL-LJ (strong leg); COD (strong leg); ConMean (weak leg); EccMean (weak leg) ↓ SL-THJ ILA; SL-CMJ ILA
				**SVS:** ↑ SL-HJ (weak leg); SL-THJ (strong leg); SL-CMJ (strong leg); COD ILA; ConMean (strong and weak leg); EccMean (strong and weak leg) = SL-HJ ILA; SL-LJ ILA; ConMean ILA; EccMean ILA; SL-HJ (strong leg); SL-THJ (weak leg); SL-CMJ (weak leg) ↓ SL-THJ ILA; SL-CMJ ILA
				**Between-group comparisons:** SVW > DVW: ConMean (weak leg); EccMean (strong and weak leg) SVW > SVS: SL-HJ (strong and weak leg) DVW > SVW: EccMean ILA; SL-THJ (weak leg); SL-CMJ (strong and weak leg) DVW > SVS: SL-HJ (strong and weak leg); SL-LJ (strong leg); SL-THJ (strong leg); SL-CMJ (strong and weak leg); COD (strong leg) SVS > SVW: ConMean ILA; SL-THJ (weak leg); COD (strong leg) SVS > DVW: ConMean (strong and weak leg); EccMean (strong and weak leg)
Gonzalo-Skok et al. [27]	*n* = 22 16 ± 1 years U-15 to U-18 highly trained basketball players VUH (starting leg = weak leg): *n* = 11 VUL (starting leg = weak leg): *n* = 11	**Test and metrics**: SL-HJ: Jump distance (cm) SL-LJ: Jump distance (cm) SL-CMJ: Jump height (cm) 180º COD: time (s) **Dominance**: Weaker leg = leg with the greater number in the same asymmetry direction (left or right) **Equation**: Limb Symmetry Index 1 = (worse leg/better leg) × 100	6 weeks (2 sessions per week) **VUH:** Side-step (1 × 6–10) Backward lunges (1 × 6–10) Crossover cutting (1 × 6–10) Landing and backward lunges 1 × 6–10) **VUL:** Lateral Squat (1 × 6–10) Defensive-like shuffling steps (1 × 6–10) Lateral crossover cutting (1 × 6–10) 90º Lunge (1 × 6–10) *Between-exercise rest:* 3 min	**VUH**: ↑ SL-CMJ ILA; SL-HJ ILA; SL-LJ ILA; SL-CMJ (right and left leg); SL-HJ (right and left leg); SL-LJ (left leg); 5-m sprint; 180º COD (right and left leg) = CMJ; SL-LJ (right leg); 10-m, 20-m, 25-m sprint; V-cut COD ↓ COD ILA
				**VUL**: ↑ SL-HJ ILA; SL-LJ ILA; SL-CMJ (left leg); SL-HJ (right and left leg); SL-LJ (right and left leg); 180º COD (right and left leg) = SL-CMJ ILA; CMJ; SL-CMJ (right leg); 5-m, 10-m, 20-m and 25-m sprint; V-cut COD ↓ COD ILA
				**Between-group comparisons:** VUH > VUL: SL-CMJ ILA; SL-CMJ (right leg); 5-m sprint VUL > VUH: SL-LJ (right and left leg)
Ujaković & Šarabon [13]	*n* = 21 19.3 ± 4.9 years Highly trained basketball players IG (nondominant-to-dominant work ratio 3:1): *n* = 10 CG (habitual weight training routine with matched volume of the IG): *n* = 11	**Test and metrics**: Single-leg isometric SQ: Peak force (N/Kg) SL-CMJ: Force impulse (Ns) COD: time (s) **Dominance**: NDL = slower side in 505 COD tests (D: stronger limb; ND: weaker limb) **Equation**: Bilateral Strength Asymmetry = [(Maximum – Minimum)/(Maximum)] × 100	8 weeks (1 session per week) **Jumping part:** SLCMJ (NDL: 4 × 6–12 / DL: 4 × 2–4) **Strength part:** Bulgarian barbell split squat (NDL: 4 × 12–6, 65–85%RM / DL: 4 × 4–2, 65–85%RM) *Between-set rest:* 3 min	**IG:** ↑ COD deficit ILA; SQ peak force (D and ND) = SQ peak force ILA; SL-CMJ force impulse ILA; SL-CMJ force impulse (D and ND); COD deficit (D and ND)
				**CG:** ↑ SQ peak force (D and ND); COD deficit (D) = SQ peak force ILA; SL-CMJ force impulse ILA; COD deficit ILA; SL-CMJ force impulse (D and ND); COD deficit (ND)
				**Between-group comparisons:** IG > CG: COD deficit ILA; SQ peak force (ND)
Zhang et al. [12]	*n* = 30 (M) 20.9 ± 1.0 years University basketball athletes IG (nondominant-to-dominant work ratio 3:1): *n* = 15 CG (regular course content): *n* = 15	**Test and metrics**: SL-CMJ: Height (cm), peak power (W), Impulse (Ns) IMTP: Peak force (N) **Dominance**: DL = left leg (inclusion criteria: “right-handed players with left-leg dominance for basketball-specific tasks”) **Equation uni asymmetry testing**: Bilateral Strength Asymmetry = (DL—NDL)/DL × 100 **Equation bil asymmetry testing**: Bilateral Asymmetry Index 1 = (DL—NDL)/(DL + NDL) × 100	10 weeks (3 sessions per week) **Resistance Training:** (DL: 1 × 6 / NDL: 3 × 6) Split squat Bulgarian split Squat Box step-up Single-leg calf raises **Plyometrics**: (DL: 1 × 12 / NDL: 3 × 12) Lunge jump Single-leg hop with back foot raise Single-leg Lateral Jump Single-leg continuous hopping *Between-set rest:* 40 s to 5 min	**IG:** ↑ SL-CMJ height ILA; SL-CMJ peak power ILA; SL-CMJ impulse ILA; IMTP ILA; CMJ; Bil horizontal jump; SQ 1RM = 10-m, 20-m sprint
				**CG**: **=** SL-CMJ height ILA; SL-CMJ peak power ILA; SL-CMJ impulse ILA; IMTP ILA; CMJ; Bil horizontal jump; 10-m, 20-m sprint
				**Between-group comparisons:** IG > CG: SL-CMJ height ILA; SL-CMJ peak power ILA; IMTP ILA; CMJ; Bil horizontal jump; SQ 1RM
**Interventions Comparing Unilateral vs. Bilateral Training Approaches**				
Ramirez-Campillo et al. [16]	*n* = 18 (M) Uni G = 17.3 ± 1.1 years Bil G = 17.6 ± 0.5 years U-19 soccer players Uni G: *n* = 9 Bil G: *n* = 9	**Test and metrics**: SL-THJ: Jump distance (m) SL-CMJ: Jump height (m) SL-SJ: Jump height (m) **Dominance**: Preferred kicking leg **Equation**: Limb Symmetry Index 1 = (worse leg / better leg) × 100	8 weeks (2 sessions per week) **Day 1:** Knee extensors (3 × 10, 70%) Knee flexors (3 × 10, 70%) 20–25 cm horizontal DJ (1 × 3–5) Horizontal jumps (1–2 × 3) **Day 2:** 20–25 cm horizontal DJ (1 × 3–5) Horizontal jumps (1–2 × 3) *Between-set rest:* 2 min	**Uni G:** ↑ SL-CMJ (D and ND); SL-SJ (D and ND); CMJ; COD; SL-THJ (D and ND); Uni crossover triple jump (D and ND) = SL-CMJ ILA; SL-SJ ILA; SL-THJ ILA; SJ; Uni crossover triple jump ILA; Bil horizontal CMJ; Bil triple horizontal CMJ; 1RM knee extension; 1RM knee flexion
				**Bil G:** ↑ Bil horizontal CMJ; Uni crossover triple jump (ND); SJ; CMJ; COD; 1RM knee extension; 1RM knee flexion = SL-CMJ ILA; SL-SJ ILA; SL-THJ ILA; Uni crossover triple jump ILA; SL-CMJ (D and ND); SL-SJ (D and ND); SL-THJ (D and ND); Uni crossover triple jump (D); Bil triple horizontal CMJ
				**Between-group comparisons:** Bil G > Uni G: 1RM knee extension; 1RM knee flexion; COD; CMJ; SJ; Bil horizontal CMJ Uni G > Bil G: SL-CMJ (D and ND); SL-SJ (D); SL-THJ (D and ND); Uni crossover triple jump (D)
Soñén et al. [26]	*n* = 22 (?) 21.8 ± 4.5 years Karate athletes Uni G: *n* = 11 Bil G: *n* = 11	**Test and metrics**: SL-HJ: Jump distance (cm) SL-CMJ: Jump height (cm) **Dominance**: Not specified **Equation**: Percentage Difference = (100 / Maximum × Minimum) × − 1 + 100	6 weeks (2 sessions per week) **Uni G:** DJ (1–3 × 10, 10–20-30 cm) **Bil G:** DJ (1–3 × 10, 20–40-60 cm) *Between-jumps rest:* 15 s *Between-set rest:* 2 min	**Uni G:** ↑ SL-HJ ILA; SL-CMJ ILA; SL-HJ D and ND; Bil horizontal jump
				**Bil G:** ↑ SL-CMJ D and ND; SL-HJ D and ND
				**Between-group comparisons:** No difference between-group
Cao et al. [11]	*n* = 66 (M) 16.1 ± 0.8 years Basketball players Uni G: *n* = 16 Bil G: *n* = 16 UniBil G: *n* = 17 CG: *n* = 17	**Test and metrics**: SL-CMJ: Peak Force (N), height (cm) Leg and hold test: Peak drop landing force (N) 5–0-5 COD: time (s) Uni isometric SQ: Peak Force (N) Uni isometric knee flexor strength: Maximum force (N) **Dominance**: Not specified **Equation**: Symmetry angle = (45° – arctan[left / right]) / 90° × 100	8 weeks (2 sessions per week) **Uni G:** Day 1: Uni horizontal jumps (2–3 × 5) Uni 3-bounce jumps (2–3 × 5) Day 2: Uni reactive pogo jumps (2–3 × 5) Uni CMJ (2–3 × 5) Uni DJ (2–3 × 5, 10 cm) **Bil G:** Day 1: Bil horizontal jumps (2–3 × 10) Bil 3-consecutive horizontal jumps (2–3 × 10) Day 2: Bil reactive pogo jumps (2–3 × 10) Bil CMJ (2–3 × 10) Bil DJ (2–3 × 10, 10 cm) **UniBil G:** Day 1: Uni horizontal jumps (2–3 × 5) Bil 3-consecutive horizontal jumps (2–3 × 10) Day 2: Bil reactive pogo jumps (2–3 × 10) Uni CMJ (2–3 × 5) Bil DJ (2–3 × 10, 10 cm) *Rest between sets and exercises:* 3 min	**Uni G:** ↑ SL-CMJ peak force ILA; SL-CMJ height ILA; leg and hold test ILA; 5–0-5 COD ILA; Uni isometric SQ ILA; Uni isometric knee flexor ILA; SL-CMJ peak force (right and left leg); SL-CMJ height (right and left leg); leg and hold test (right and left leg); 5–0-5 COD (right and left leg); Uni isometric SQ (right and left leg); Uni isometric knee flexor (right and left leg)
				**Bil G:** ↑ SL-CMJ height ILA; SL-CMJ height (left leg); leg and hold test (right and left leg); 5–0-5 COD (right and left leg); Uni isometric SQ (left leg) = SL-CMJ peak force ILA; leg and hold test ILA; 5–0-5 COD ILA; Uni isometric knee flexor ILA; SL-CMJ peak force (right and left leg); SL-CMJ height (right leg); Uni isometric SQ (right leg); Uni isometric knee flexor (right and left leg) ↓ Uni isometric SQ ILA
				**UniBil G:** ↑ SL-CMJ height ILA; 5–0-5 COD ILA; Uni isometric SQ ILA; SL-CMJ peak force (right and left leg); SL-CMJ height (left leg); leg and hold test (right and left leg); 5–0-5 COD (right and left leg); Uni isometric SQ (right and left leg); Uni isometric knee flexor (right and left leg) = SL-CMJ peak force ILA; leg and hold test ILA; Uni isometric knee flexor ILA; SL-CMJ height (right leg)
				**CG:** ↑ SL-CMJ height ILA; SL-CMJ height (left leg) = SL-CMJ peak force ILA; leg and hold test ILA; 5–0-5 COD ILA; SL-CMJ peak force (right and left leg); SL-CMJ height (right leg); leg and hold test (right and left leg); 5–0-5 COD (right and left leg); Uni isometric SQ (right leg); Uni isometric knee flexor (right and left leg) ↓ Uni isometric SQ ILA; Uni isometric knee flexor ILA; Uni isometric SQ (left leg)
				**Between-group comparisons:** Uni G > Bil G: SL-CMJ peak force (left leg); leg and hold test (right and left leg); 5–0-5 COD (left leg); Uni isometric SQ (right and left leg); Uni isometric knee flexor (right and left leg) Uni G > UniBil G: SL-CMJ height (left leg); leg and hold test (left leg); Uni isometric SQ (left leg) Uni G > CG: leg and hold test ILA; Uni isometric SQ ILA; Uni isometric knee flexor ILA; SL-CMJ peak force (right and left leg); SL-CMJ height (right and left leg); leg and hold test (right and left leg); 5–0-5 COD (right and left leg); Uni isometric SQ (right and left leg); Uni isometric knee flexor (right and left leg) UniBil G > CG: leg and hold test (right and left leg); 5–0-5 COD (right and left leg); Uni isometric SQ (left leg); Uni isometric knee flexor (right and left leg) Bil G > CG: Uni isometric SQ (left leg)
Li et al. [20]	*n* = 20 (M) 20.8 ± 1.9 years University basketball players Uni G: *n* = 10 Bil G: *n* = 10	**Test and metrics**: SL-CMJ: Jump height (cm) **Dominance**: Not specified **Equation**: Bilateral Asymmetry Index 2 = (2 ×|left – right|/(left + right)) × 100	8 weeks (2 sessions per week) Vertical jump DJ (Uni G: 15 cm, Bil G: 30 cm) Lateral hurdle jump (Uni G: 15 cm, Bil G: 30 cm) Hurdle jump forward (Uni G: 15 cm, Bil G: 30 cm) Standing long jump Uni G: 2 × 4 Bil G: 2 × 8 Phase 1 (week 1–2): Jumps and freezing Phase 2 (week 3–4): Ankle jumps before jumps Phase 3 (week 5–6): Consecutive jumps *Between-set rest:* 1 min *Between-exercise rest:* 2 min	**Uni G:** ↑ SL-CMJ (right and left leg); CMJ; RSI (right and left leg); RSI Bil; 505-COD (right and left leg); 10-m sprint; COD deficit (right and left leg); V-cut COD = SL-CMJ ILA;
				**Bil G**: ↑ CMJ; RSI Bil; 505-COD (right and left leg); COD deficit (left leg); V-cut COD = SL-CMJ ILA; SL-CMJ (right and left leg); RSI (right and left leg); 10-m sprint; COD deficit (right leg)
				**Between-group comparisons:** Uni G > Bil G: SL-CMJ (left leg); 505-COD (right and left leg); COD deficit (right and left leg); V-cut COD

*1RM* one-repetition maximum, *Bil* bilateral, *Bil G* bilateral group, *CG* control group, *CMJ* countermovement jump, *COD* change of direction, *ConMean* mean concentric power, *ConPeak* peak concentric power, *DL* dominant limb, *DJ* drop jump, *DVW* double volume on weaker leg, weaker leg first, *EccMean* mean eccentric power, *EccPeak* peak eccentric power, *G* group, *IG* intervention group, *ILA* interlimb asymmetry, *IMTP* isometric mid-thigh pull, *LSI* limb symmetry index, *M* male, *NDL* nondominant limb, *RSI* reactive strength index, *SJ* squat jump, *SL-CMJ* single leg countermovement jump, *SL-HJ* single leg horizontal jump, *SL-LJ* single leg lateral jump, *SL-SJ* single leg squat jump, *SL-THJ* single leg triple horizontal jump, *SQ* squats, *SVS* same volume, stronger leg first, *SVW* same volume, weaker leg first, *Uni* unilateral, *Uni G* unilateral group, *UniBil G* combined unilateral and bilateral group, *VUH* variable unilateral horizontal movements, *VUL* variable unilateral lateral movements, *↑,* improved or better performance within group or compared to another group; *↓,* worsened or poorer performance; =*,* no difference

ILA were assessed using a variety of performance tests, including unilateral jump tests such as the SL-CMJ, SL-HJ, triple horizontal jump (SL-THJ), lateral jump (SL-LJ), and squat jump (SL-SJ), as well as COD tests, power output during the lateral squat exercise, isometric strength tests (unilateral isometric squat and unilateral isometric knee flexor strength), and the leg and hold test.

#### Interventions Applying Different Training Stimuli Between Limbs

Seven of the 11 included studies applied asymmetric training protocols aimed at reducing ILA. Three studies [10, 14, 18] divided participants into three groups: one performed unilateral exercises with equal volume on both legs, always starting with the weaker leg (SVW); another applied double training volume to the weaker leg, also initiating with that leg (DVW); and a third used equal volume but began with the stronger leg (SVS). Gonzalo-Skok et al. [17] compared unilateral and bilateral training, with the unilateral group always starting exercises with the weaker leg, despite matched training volume. Another study by Gonzalo-Skok et al. [27] compared variable unilateral horizontal (VUH) and variable unilateral lateral (VUL) movement patterns, both beginning with the weaker leg. Lastly, two studies evaluated training protocols with increased volume for the weaker limb, contrasting them with either matched-volume conditions [13] or regular course content [12].

Training protocols varied across studies and included plyometric and weight lifting [12, 13, 17], lateral squats using conical pulley devices [10, 14] or vibration platforms [18], and eccentric-overload training [27]. Methods for determining weakness varied across studies. Most defined the weaker leg based on performance in unilateral tests, such as jumps [10, 14, 18], unilateral squats [17], or COD assessments [13], or by identifying the side with the greatest and most consistent asymmetry [27]. In contrast, one study [12] determined dominance based on sport-specific inclusion criteria, selecting right-handed players with left-leg dominance (i.e., preferred takeoff leg) for basketball tasks.

#### Interventions Comparing Unilateral vs. Bilateral Training Approaches

Four of the 11 included studies compared unilateral and bilateral training approaches. Three studies [16, 20, 26] allocated participants into unilateral and bilateral training groups, although one study [11] included additional groups combining unilateral and bilateral exercises, along with a control group. The training interventions primarily consisted of plyometric jump programs, with one study combining weight lifting and plyometric training [16]. Regarding dominance assessment, most studies did not report any method for identifying the stronger or weaker limb; only Ramirez-Campillo et al. [16] defined the dominant leg as the preferred kicking limb (i.e., preference-based dominance).

### Risk of Bias Assessment

**Fig. 2:**
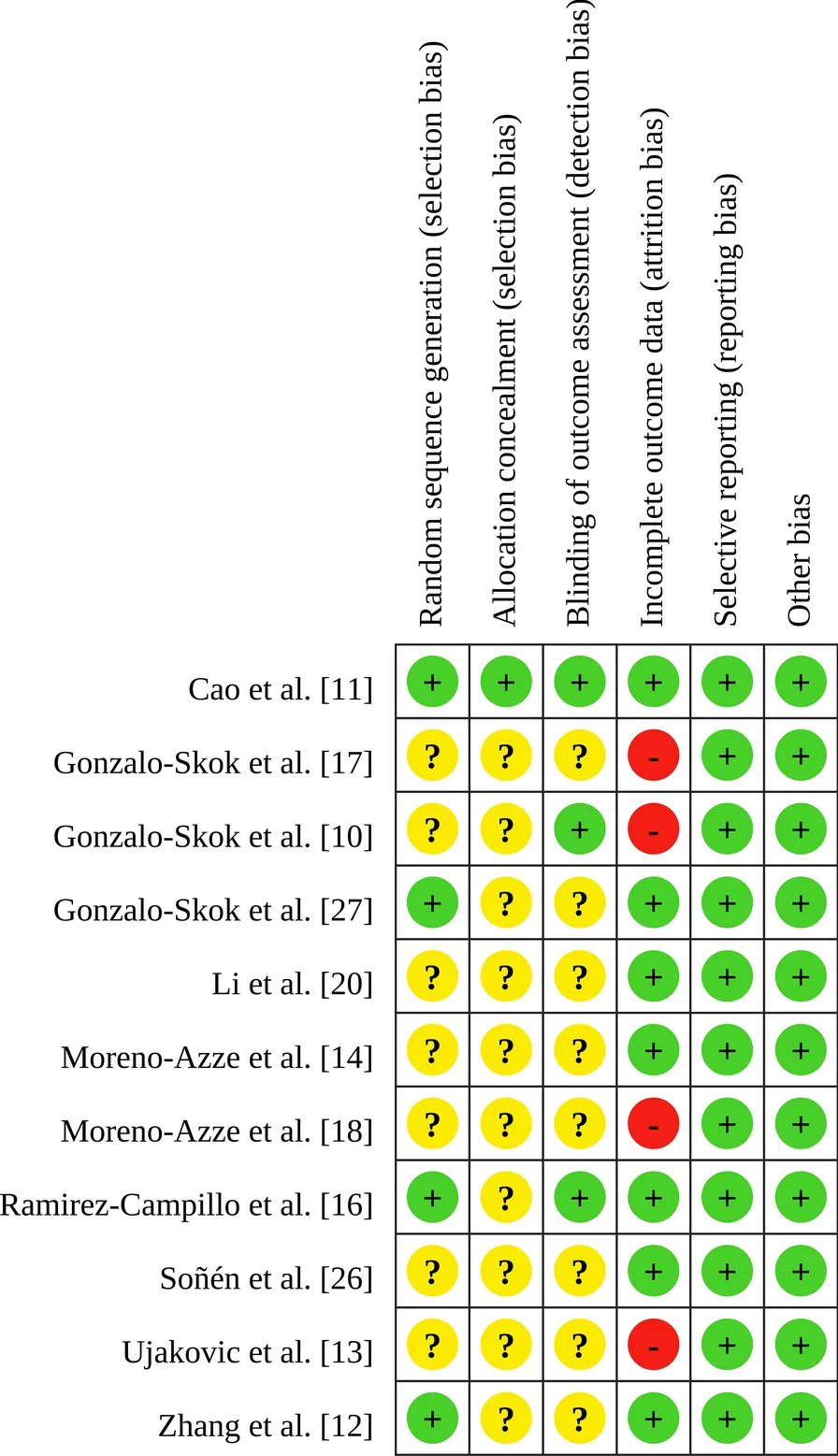
Risk of bias assessment by domain and study. The risk of bias is visually represented using colors: green (low risk), yellow (unclear risk), and red (high risk)

Based on the assessment of selection bias, only four studies were judged as having a low risk of bias for random sequence generation. The remaining seven studies were classified as having an unclear risk, primarily due to the nature of their randomization procedures. Specifically, randomization in these studies was conducted based on ranked physical performance [10, 14, 17, 18, 26], age and the magnitude of COD deficit [13], or stratification by playing position [20], all of which may introduce potential bias. Regarding allocation concealment, only the study by Cao et al. [11] clearly reported adequate procedures, although the others lacked sufficient detail and were therefore rated as unclear risk. In terms of detection bias, only three studies [10, 11, 16] clearly reported blinding of outcome assessment, as they explicitly stated that the assessors were unaware of group allocation. The remaining studies did not provide this information and were therefore rated as unclear risk. Regarding attrition bias, seven of the 11 studies were judged as low risk. The remaining four [10, 13, 14, 17] had substantial participant dropout (ranging from ~20% to over 40%), which could potentially impact the reliability of the results, and were rated as high risk. Concerning reporting bias, all studies were rated as low risk. Finally, all studies were judged as low risk for other sources of bias. The risk of bias assessment for each included study is presented in Fig. 2.

## Discussion

This systematic review aimed to evaluate the effectiveness of training interventions for reducing ILA and enhancing athletic performance. Specifically, it compared asymmetry-focused programs based on baseline ILA values with general training approaches, and unilateral versus bilateral training protocols. Eleven studies involving 297 athletes met the inclusion criteria. Although most interventions led to performance improvements, reductions in ILA were more consistently observed in programs that individualized the training stimulus. Nonetheless, methodological differences across studies make it difficult to draw firm conclusions.

### Interventions Prescribed According to Baseline ILA

#### Starting with the Weaker Leg

Several studies have examined whether initiating unilateral exercises with the weaker leg leads to significant reductions in ILA and superior athletic adaptations. In Gonzalo-Skok et al. [17], a unilateral training group that started each set with the weaker leg showed greater improvements in the concentric peak power of an incremental unilateral squat asymmetry test, as well as improvements in bilateral squat deficit, compared to a bilateral training group. Similarly, Gonzalo-Skok et al. [10] reported a significantly greater reduction in SL-HJ asymmetry for the group that started with the weaker leg compared to the group that started with the stronger leg. These results are supported by Moreno-Azze et al. [14], where starting with the weaker leg also led to a greater reduction in concentric peak power asymmetry than starting with the stronger leg. Conversely, in Moreno-Azze et al. [18], no meaningful between-group differences in ILA were observed across any of the assessed tests, indicating that this approach may not always produce superior outcomes. Although Gonzalo-Skok et al. [27] did not include a comparator group with a different limb order or bilateral training, both groups, which started with the weaker leg, showed reductions in jump asymmetries. The authors suggested that this sequencing strategy may be particularly effective, showing more favorable results than protocols that did not control limb order.

In terms of physical performance, Gonzalo-Skok et al. [17] found that the unilateral group that started with the weaker leg outperformed the bilateral group in the CMJ, 180º COD with the left leg, and in incremental unilateral squat concentric peak power with both legs, highlighting a broad performance advantage of the unilateral approach that started with the weaker leg. In Gonzalo-Skok et al. [10], the groups that either applied double training volume to the weaker leg or began each set with the weaker leg showed superior gains compared to the group that started with the stronger leg. Moreno-Azze et al. [14] also reported that beginning sets with the weaker leg resulted in better outcomes than starting with the stronger leg across multiple power measures in the lateral squat exercise. In another study by Moreno-Azze et al. [18], only SL-HJ performance (strong and weak leg) was significantly greater in the group that started with the weaker leg compared to the group that started with the stronger one. Furthermore, this same group also outperformed the group that started and applied double training volume to the weaker leg in concentric mean power (weak leg) and eccentric mean power (both legs) in the lateral squat exercise. Finally, in Gonzalo-Skok et al. [27], the horizontal group showed greater improvements in SL-CMJ (right leg) and 5-m sprint, although the lateral group improved more in SL-LJ in both legs.

Taken together, the evidence suggests that starting unilateral exercises with the weaker leg may provide modest but consistent benefits in both reducing ILA and enhancing physical performance. It nonetheless appears to be a simple, low-risk approach that can be easily implemented in training programs, as it requires no additional equipment or time and is unlikely to produce adverse effects. This approach represents a subtle yet meaningful modification in training prescription, as it may allow the weaker limb to receive a superior neuromuscular stimulus although still in an unfatigued state. Conversely, consistently initiating training with the stronger leg may inadvertently reinforce existing ILA, compounding the asymmetries already induced by sport-specific practice [19]. Therefore, the targeted overload applied to the weaker limb under non-fatigued conditions may represent a window of opportunity to reduce the performance gap with its contralateral counterpart [19], thereby possibly attenuating ILA and enhancing physical performance in both legs. Its potential usefulness, combined with its practicality, makes it a promising tool in the context of individualized athletic training, although further research is needed to confirm its effectiveness across different populations.

#### Starting and Applying Double Volume to the Weaker Leg

Regarding protocols that applied double training volume and started each set with the weaker leg, three studies have reported favorable effects on ILA compared to other training prescriptions. In Gonzalo-Skok et al. [10], the group that applied double volume and started with the weaker leg showed significantly greater reductions in SL-THJ asymmetry than both the group that started with the weaker leg and the group that started with the stronger leg. This finding was reinforced in Moreno-Azze et al. [14], where the group that applied double volume and started with the weaker leg achieved a more pronounced reduction in eccentric peak power asymmetry compared to the other training conditions. However, these two studies were conducted on the same sample, so their findings should be interpreted with caution. Additionally, Moreno-Azze et al. [18] reported that the group applying double volume and starting with the weaker leg was more effective than the group that started with the weaker leg in reducing eccentric mean power asymmetry.

In terms of physical performance, Gonzalo-Skok et al. [10] reported that the group starting and applying double volume to the weaker leg showed greater improvements in jump performance than the group that started with the stronger leg. Both this group and the group that started with the weaker leg outperformed the group that started with the stronger leg across several jump tests. In Moreno-Azze et al. [14], the group that started and applied double volume to the weaker leg achieved greater improvements in power output compared to both the group that started with the weaker leg and the group that started with the stronger leg. Finally, Moreno-Azze et al. [18] also found that the group applying double volume and starting with the weaker leg produced greater improvements than the other training groups across a wide range of jump, COD, and power performance tests.

Although the current findings indicate that this methodology could be a promising strategy to improve both ILA and physical performance, only three studies have explored its effects. Moreover, two of them [10, 14] were conducted on the same sample, which may limit the applicability of their results. Interestingly, applying a higher training volume to the weaker limb often led to greater improvements than simply starting with it, suggesting that increased stimulus of the weaker limb may enhance adaptations. This could be attributed to the greater progressive overload imposed on the weaker limb, which may accelerate its neuromuscular adaptations [28] and, over time, bring its performance capacity closer to that of the stronger leg. In this sense, doubling the training volume applied to the weaker limb may further exploit the window of opportunity previously described [19], potentially yielding more pronounced reductions in ILA and greater improvements in physical performance. From a practical standpoint, this approach appears safe and potentially effective, as it consistently produced positive outcomes in both asymmetry reduction and performance enhancement, without reported adverse effects. Nevertheless, further studies are needed to confirm these initial findings and to better understand the specific mechanisms and conditions under which this method is most beneficial.

#### Starting with the Stronger Leg

The available evidence does not support starting unilateral exercises with the stronger leg as an effective strategy for reducing ILA. Across the three studies that included this approach [10, 14, 18], no consistent benefits were observed in ILA outcomes. In fact, in most comparisons, starting with the stronger leg showed smaller or no improvements compared to starting with the weaker leg and starting and applying double volume to the weaker leg. The only exception was found in Moreno-Azze et al. [18], where the group that started with the stronger leg achieved a greater reduction in concentric mean power asymmetry than the group that started with the weaker leg.

Regarding physical performance, the group that started with the stronger leg showed limited evidence of superiority across the included studies. In both Gonzalo-Skok et al. [10] and Moreno-Azze et al. [14], the group that used this approach did not outperform any other training group in performance outcomes. However, Moreno-Azze et al. [18] reported a few isolated advantages for the group that started with the stronger leg, including greater improvements than starting with the weaker leg in SL-THJ (weak leg) and COD (strong leg). Additionally, starting with the stronger leg showed better results than starting and applying double volume to the weaker leg in some isolated power measures.

Despite these findings, the lack of consistent benefits across studies suggests that starting with the stronger leg may not be a reliable strategy for enhancing physical performance. From a mechanistic standpoint, initiating training with the stronger leg may not provide sufficient additional stimulus to the weaker limb, as the latter would be trained under conditions of accumulated fatigue, potentially limiting the quality of its neuromuscular recruitment and, consequently, its adaptive response. In this sense, this approach may inadvertently widen the existing performance gap between limbs, which could partly account for the limited improvements observed in both ILA and physical performance. It is worth noting that the two studies that did not report favorable results for this approach [10, 14] were conducted using the same participant sample, which may partially explain the similarity in their outcomes. Given the limited number of studies and the methodological differences between them, further research is needed to clarify whether initiating training with the stronger limb offers any meaningful advantages in terms of performance or ILA reduction.

#### Triple Volume Applied to the Weaker Leg

Two recent studies have explored the effects of applying triple training volume to the weaker leg on ILA, with preliminary but encouraging results. Ujaković & Šarabon [13] reported that the intervention group showed a greater reduction in COD deficit asymmetry compared to a control group that received a matched training volume. Similarly, Zhang et al. [12] observed improvements in several ILA variables, including SL-CMJ height, SL-CMJ peak power, and isometric mid-thigh pull (IMTP) asymmetry.

In terms of physical performance, both studies reported positive outcomes compared to control groups. Ujaković & Šarabon [13] found that the intervention group achieved greater gains in peak force during the squat exercise with the weaker leg. In line with this, Zhang et al. [12] reported improvements in several performance variables, including CMJ height, bilateral horizontal jump distance, and squat one-repetition maximum (1RM).

This current evidence suggests that applying a higher training volume to the weaker leg may lead to additional improvements compared to control groups. As previously discussed in the context of double-volume protocols, these findings could similarly be attributed to the greater progressive overload imposed on the weaker limb [28]. This may accelerate its neuromuscular adaptations and, over time, bring its performance capacity closer to that of the stronger leg, further exploiting the window of opportunity previously described [19]. Only two studies have investigated this methodology, which limits the strength of the available evidence. Moreover, it is important to note that in Zhang et al. [12], the control group continued with their regular training routine, but the content of that routine was not clearly specified. This lack of detail suggests that the intervention and control groups likely differed in both training volume and content, which may partly explain the superior results observed in the intervention group. Therefore, further research is needed to confirm the effectiveness of triple-volume prescriptions and to determine under which conditions they might provide meaningful benefits in both ILA reduction and performance enhancement.

### Interventions Comparing Unilateral versus Bilateral Training Approaches

The evidence comparing unilateral and bilateral training approaches in relation to ILA is currently limited and inconclusive. Three of the four available studies [16, 20, 26] did not report significant between-group differences in ILA outcomes. In contrast, Cao et al. [11] found that the unilateral training group achieved greater reductions in asymmetry compared to a control group in several tests, including the leg and hold test, unilateral isometric squat, and unilateral isometric knee flexor assessments.

The comparison between unilateral and bilateral training protocols has yielded mixed results regarding physical performance outcomes. In Ramirez-Campillo et al. [16], the bilateral group outperformed the unilateral group in a range of bilateral tasks, whereas the unilateral group showed superior improvements in single-limb performance tests. Soñén et al. [26] found no significant between-group differences in any performance variables. In contrast, Cao et al. [11] reported a comprehensive set of improvements in the unilateral group across jump, COD, and strength tests compared to the bilateral, combined and control groups. The combined unilateral and bilateral group also outperformed the control group in several tests, while the bilateral group showed limited advantages over the control. Finally, Li et al. [20] observed greater improvements in the unilateral than in the bilateral group across jumps and COD performance tests.

Although unilateral training appears to offer some advantages over bilateral approaches in unilateral performance tests and ILA reduction, the overall evidence remains inconclusive. Only one of the four available studies reported clear benefits in favor of unilateral training for reducing ILA, while others failed to demonstrate significant between-group differences. Regarding performance outcomes, unilateral training appeared to produce superior gains in single-limb tests, while bilateral training showed greater improvements in bilateral tasks, highlighting a task-specific response pattern as observed in previous studies [29, 30]. Additionally, in studies where improvements were observed, these were often limited to one leg or to certain isolated tests, which limits the generalizability of the findings. In contrast, the recent systematic review and meta-analysis by Sun et al. [8] provides more robust evidence, concluding that unilateral plyometric training effectively reduces lower ILA in athletes, whereas bilateral and complex training do not yield significant effects. This reinforces the potential value of unilateral training, particularly plyometric exercises, as a practical strategy for targeting ILA. A possible explanation for the potential benefits of unilateral training lies in the principle of training specificity. By training each limb independently, unilateral exercises may promote greater sport-specific adaptations, given that the majority of athletic movements, such as sprinting, jumping, or COD, are predominantly unilateral in nature [31]. Furthermore, the progressive overload applied to each limb individually may elicit superior neuromuscular adaptations compared to bilateral training, where the stronger limb may partially compensate for deficits in the weaker one [28]. However, the mechanisms underlying these adaptations remain largely speculative, and further high-quality studies are needed to determine the conditions under which unilateral approaches may offer consistent advantages over bilateral training.

### Limitations

Several methodological and conceptual limitations should be acknowledged when interpreting the findings of this systematic review. First, the definition and identification of limb dominance varied across studies, with some relying on self-reported preference and others using performance-based metrics. However, these metrics also differed between studies, and no standardized methodology exists for determining dominance in specific movement tasks, such as jumping or strength assessments. Additionally, there is no consensus on how to calculate ILA. Although some authors have proposed standardized equations [4, 6], variability in the formulas used and the direction of comparison remains a common issue. Furthermore, none of the included studies reported whether baseline asymmetries exceeded the coefficient of variation (CV) of the respective assessment tools, an important criterion to confirm that observed asymmetries represent genuine inter-limb differences rather than measurement noise. Although some studies applied the smallest worthwhile change (SWC) to evaluate the practical significance of post-intervention modifications, this approach remains methodologically insufficient in the absence of prior baseline verification, constituting a notable gap in the current body of evidence. Beyond these methodological concerns, the current body of evidence is limited by the small number of studies per intervention type, reduced sample sizes, and heterogeneous training protocols. Additionally, two included studies were derived from the same sample and were therefore counted only once when reporting the total number of participants [10, 14]. Although the present review was based on a qualitative synthesis rather than a meta-analysis, the inclusion of multiple publications from the same cohort may have led to a slight overrepresentation of their findings in the narrative interpretation of the evidence. These factors limit the ability to generalize the results and identify the most effective approaches for reducing ILA and improving athletic performance. Furthermore, the heterogeneity of participant samples across studies, including differences in sex, competitive level, age, and sport, may limit the comparability of findings and the generalizability of conclusions to specific athletic populations.

### Practical Applications and Future Directions

Certain training strategies, such as starting unilateral exercises with the weaker leg or applying more volume to that limb, appear to be safe, easy to implement, and potentially beneficial for reducing ILA and enhancing performance. These methods require no additional equipment or time, making them viable for application in most athletic contexts. However, their success depends on establishing an accurate criterion for identifying the weaker limb. Specifically, the stronger limb should be identified as the limb demonstrating superior performance in the assessed task, rather than relying on subjective preference or limb-use questionnaires. We recommend using performance-based methods aligned with the specific task being trained or assessed (e.g., horizontal jump performance to establish dominance in horizontal jumping tasks). Based on this, training interventions should be individualized to each athlete’s asymmetry profile and performance needs. Given that ILA is inherently individual and task-dependent, group-level analyses may not fully capture the asymmetry profile of each athlete. Accumulating more consistent evidence across different sports, populations, and outcome measures will be essential to clarify whether interventions specifically designed to reduce ILA truly lead to meaningful improvements in athletic performance. Additionally, future research should verify whether baseline asymmetries exceed the CV of the assessment tool before intervening, in order to confirm that they reflect true inter-limb differences rather than measurement noise. Similarly, post-intervention changes should be evaluated against this threshold to distinguish real improvements from measurement variability.

## Conclusion

This systematic review suggests that certain training strategies, such as initiating unilateral exercises with the weaker leg or applying greater training volume to it, may be promising methods for reducing ILA and enhancing athletic performance. These asymmetry-focused approaches appear more effective than conventional, non-targeted unilateral or bilateral training, and are generally practical, low-risk, and applicable in athletic settings. However, the current body of evidence is insufficient to draw definitive conclusions, as most findings stem from a limited number of studies with small sample sizes and considerable methodological variability. Therefore, while asymmetry-focused interventions represent a viable and potentially superior option for reducing ILA, further high-quality research is needed to confirm their effectiveness and support the development of evidence-based guidelines for their implementation.

## Supplementary Information

Supplementary Material 1.

## Data Availability

The datasets generated and/or analyzed during the current review are available from the corresponding author upon reasonable request.

## Abbreviations

1RM: One-repetition maximum
Bil: Bilateral
Bil G: Bilateral group
CG: Control group
CMJ: Countermovement jump
COD: Change of direction
ConMean: Mean concentric power
ConPeak: Peak concentric power
DL: Dominant limb
DJ: Drop jump
DVW: Double volume on weaker leg weaker leg first
EccMean: Mean eccentric power
EccPeak: Peak eccentric power
G: Group
IG: Intervention group
ILA: Interlimb asymmetry
IMTP: Isometric mid-thigh pull
LSI: Limb symmetry index
M: Male
NDL: Nondominant limb
RSI: Reactive strength index
SJ: Squat jump
SL-CMJ: Single leg countermovement jump
SL-HJ: Single leg horizontal jump
SL-LJ: Single leg lateral jump
SL-SJ: Single leg squat jump
SL-THJ: Single leg triple horizontal jump
SQ: Squats
SVS: Same volume stronger leg first
SVW: Same volume weaker leg first
Uni: Unilateral
Uni G: Unilateral group
UniBil G: Combined unilateral and bilateral group
VUH: Variable unilateral horizontal movements
VUL: Variable unilateral lateral movements
