# Supplementary material for: Are Asymmetry-Focused Training Interventions More Effective than General Programs in Reducing Interlimb Asymmetries and Improving Athletic Performance? A Systematic Review
Source: Sports Med Open. 2026 Oct 13;12:156. doi: 10.1186/s40798-026-01119-5 (PMC13661881; doi:10.1186/s40798-026-01119-5)
Supplement: Supplementary file 1 [file 40798_2026_1119_MOESM1_ESM.docx]

**Supplementary Material 1. Full Search Strategy used in each database:**

**PubMed**: ("unilateral training"[Title/Abstract] OR "bilateral training"[Title/Abstract] OR "resistance training"[Title/Abstract] OR "strength training"[Title/Abstract] OR "plyometric exercise"[Title/Abstract] OR "plyometric training"[Title/Abstract] OR "training intervention"[Title/Abstract] OR "exercise intervention"[Title/Abstract] OR "exercise program"[Title/Abstract] OR "exercise training"[Title/Abstract] OR "neuromuscular training"[Title/Abstract] OR "conditioning program"[Title/Abstract]) AND ("physical functional performance"[Title/Abstract] OR "functional performance"[Title/Abstract] OR "physical performance"[Title/Abstract] OR "athletic performance"[Title/Abstract] OR "sports performance"[Title/Abstract] OR "single leg"[Title/Abstract] OR strength[Title/Abstract] OR power[Title/Abstract] OR speed[Title/Abstract] OR jump[Title/Abstract] OR sprint[Title/Abstract] OR "change of direction"[Title/Abstract] OR agility[Title/Abstract]) AND ("asymmetr*"[Title/Abstract] OR "inter-limb asymmetry"[Title/Abstract] OR "inter-limb difference"[Title/Abstract] OR "between-limb asymmetry"[Title/Abstract] OR "between-limb imbalance"[Title/Abstract] OR "side-to-side difference"[Title/Abstract] OR "left-right difference"[Title/Abstract] OR "limb asymmetry"[Title/Abstract] OR "limb-dominance"[Title/Abstract] OR "lower-limb asymmetry"[Title/Abstract] OR "limb discrepancy"[Title/Abstract] OR "dominant limb"[Title/Abstract] OR "non-dominant limb"[Title/Abstract])

**Web of Science**: ((TS=((“unilateral training” OR “bilateral training” OR “resistance training” OR “strength training” OR "plyometric exercise" OR “plyometric training” OR “training intervention” OR “exercise intervention” OR “exercise program” OR “exercise training” OR “neuromuscular training” OR “conditioning program”))) AND TS=(("physical functional performance" OR “functional performance” OR “physical performance” OR “athletic performance” OR “sports performance” OR “single leg” OR strength OR power OR speed OR jump OR sprint OR “change of direction” OR agility))) AND TS=((“asymmetr*” OR “inter-limb asymmetry” OR “inter-limb difference” OR “between-limb asymmetry” OR “between-limb imbalance” OR “side-to-side difference” OR “left-right difference" OR “limb asymmetry” OR “limb-dominance” OR “lower-limb asymmetry” OR “limb discrepancy” OR “dominant limb” OR “non-dominant limb”))

**SportDISCUS**: XB ((“unilateral training” OR “bilateral training” OR “resistance training” OR “strength training” OR "plyometric exercise" OR “plyometric training” OR “training intervention” OR “exercise intervention” OR “exercise program” OR “exercise training” OR “neuromuscular training” OR “conditioning program”)) AND XB (("physical functional performance" OR “functional performance” OR “physical performance” OR “athletic performance” OR “sports performance” OR “single leg” OR strength OR power OR speed OR jump OR sprint OR “change of direction” OR agility)) AND XB ((“asymmetr*” OR “inter-limb asymmetry” OR “inter-limb difference” OR “between-limb asymmetry” OR “between-limb imbalance” OR “side-to-side difference” OR “left-right difference" OR “limb asymmetry” OR “limb-dominance” OR “lower-limb asymmetry” OR “limb discrepancy” OR “dominant limb” OR “non-dominant limb”))
